# Probiotics potentials in mitigating coronavirus disease (COVID-19) pandemic

**DOI:** 10.11604/pamj.2021.38.186.27953

**Published:** 2021-02-18

**Authors:** Rine Christopher Reuben, Makwin Danladi Makut, Lillian Yami Adogo

**Affiliations:** 1 German Centre for Integrative Biodiversity Research (iDiv), Halle-Jena-Leipzig, Germany,; 2 Department of Microbiology, Nasarawa State University, Keffi, Nigeria,; 3 Department of Biological Sciences, Bingham University, Karu, Nigeria

**Keywords:** COVID-19, pandemic, probiotics, microbiota, mitigate, prevention

## Abstract

Despite the adoption and use of different infection prevention and control measures, the coronavirus disease (COVID-19) pandemic keeps surging on with globally increasing morbidities and mortalities. The lack of a specific therapeutic intervention against COVID-19 warrants the use of non-conventional potent alternatives. In recent times, probiotics have shown to mitigate numerous health challenges, including animal and human infectious diseases through competitive exclusion or antagonism of pathogens, modulation of host-microbiota, secretion of antimicrobial compounds and stimulation of immune responses. The presentation of COVID-19 as severe respiratory distress leading to gastrointestinal tract involvement could be mitigated through probiotics administration which beneficially modulates the microbiota and immune responses with an attendant reduction in morbidities, hence curtailing the COVID-19 pandemic.

## Commentary

In December 2019, the world was notified of a severe respiratory disease outbreak in the city of Wuhan, the capital of Central China´s Hubei province. This outbreak was identified to be caused by a novel coronavirus (2019-nCoV) or severe acute respiratory syndrome coronavirus 2 (SARS-CoV-2) which soon thereafter circulated worldwide causing the present coronavirus disease 2019 (COVID-19) pandemic, an immense global health threat with 93,956,883 cases and 2,029,084 deaths confirmed globally as of 19^th^ January 2021 [1]. The presentation of COVID-19 is characterized with a spectrum of disease severity which occurs in infected individuals, primarily ranging from mild respiratory and non-specific flu-like symptoms to fever, cough and fatigue and to severe life-threatening pneumonia, acute respiratory distress syndrome (ARDS) and multiple organ failure [2]. Also, COVID-19 infected individuals often show gastrointestinal diseases including diarrhoea and dysbiosis. Although the transmission of SARS-CoV-2 is thought to be primarily through respiratory droplets and contact routes, the prolonged isolation of SARS-CoV-2 in stool samples of active and non-active cases depicts the contribution and involvement of the human gut in the COVID-19 pathogenesis [2].

The lack of specific therapeutic interventions and vaccines against COVID-19 has led to the rapidly increasing number of COVID-19 cases globally thus, exerting significant risk to overburdened healthcare systems of the countries of the world. Furthermore, different infection prevention and control measures including hygiene, social distancing, lockdown, use of face mask, screening, isolation and quarantine have been adopted to curtail COVID-19 pandemic globally [1]. Nevertheless, despite these intervention strategies, COVID-19 still rages like wildfire with an increasing number of cases and death reported throughout the world. So far, COVID-19 cases are managed through supportive care, provision of oxygen and fluids and antibiotics (especially for diarrhoea). The use of hydroxychloroquine, an anti-malarial drug often with a second-generation macrolide (especially azithromycin) has gained preeminence in the management of COVID-19, despite non-conclusive clinical evidence supporting their use. Also, early epidemiological data has shown significant potentials in the use of remdesivir, an anti-viral drug proven to be effective against Ebola and ritonavir, an anti-HIV drug known to be a protease inhibitor [3]. While the identification and randomized controlled trials (RCTs) of several therapeutic interventions including antivirals and immunotherapies are underway, the development of effective and safe vaccines against COVID-19 will undoubtedly take several months and/or years. Therefore, there is immense pressure for the identification of other therapeutic and/or prophylactic strategies to urgently mitigate the COVID-19 pandemic. In recent decades, there has been a growing interest in the use of microbial strains found in our microbiota and/or exogenous microorganisms as probiotics for the treatment and prevention of both infectious and non-infectious diseases. Probiotics are viable microorganisms that confer health benefits to the host when administered in adequate amounts [4].

Strains of lactic acid bacteria (LAB) especially *Lactobacillus* and *Bifidobacterium* have been traditionally used as probiotics to mitigate gastrointestinal tract and vaginal infections and also to reduce pathogen colonization [4]. However, recent studies have indicated the protective effect of some strains of LAB, *Corynebacterium* and *Dolosigranulum spp*. in the treatment of respiratory tract infections. More so, the presence of *Corynebacterium* and *Dolosigranulum spp*. as prevalent members of the respiratory tract microbiota have consistently been correlated with improved respiratory health, for example, lower rates of viral infections, especially in children [5]. Probiotics generally exert beneficial effects through competitive exclusion or antagonism of pathogens, modulation of host-microbiota, secretion of antimicrobial compounds and stimulation of immune responses [4].

Over 90% of respiratory tract infections (RTIs) (especially in the upper region) are caused by viruses. The beneficial role played by probiotics in the mitigation of RTIs caused by a wide variety of pathogens including viruses have been reported in different studies. Apart from being a safe and natural intervention, the antiviral activity of probiotics and their metabolites could lead to their wide acceptance as the most suitable treatment for COVID-19 if proven efficacious. Also, the immunomodulatory activity exerted by probiotics enhances host immune defenses against different viruses, including the induction of immunoglobulins, macrophages, natural killer cells, interleukins and T-helper cells action [4]. The production of different molecules with antibacterial and antiviral activity including organic acids (such as acetic and lactic acids), bacteriocins, hydrogen peroxide (H_2_O_2_), short-chain fatty acids (SCFAs), polysaccharides and several other bacteriocin-like molecules by probiotics and their selective stimulation of the growth of other beneficial microorganisms also endears their usage as a sustainable therapeutic measure against infectious diseases including COVID-19.

The efficacy of probiotics in mitigating and reducing both the duration and incidence of viral RTIs have been previously reported in separate meta-analyses. In two RCTs, patients with severe pneumonia placed on mechanical ventilation improved significantly after the administration of probiotics composed of strains of *Bacillus subtilis, Enterococcus faecalis* and *L. rhamnosus* GG [6]. Furthermore, in another RCT involving 94 preterm infants, Luoto *et al*. [7] reported a lowered incidence of RTI associated with clinically defined virus by 2- to 3-fold compared to placebo after the use of probiotic *L. rhamnosus*GG or prebiotic mixture containing galactooligosaccharide and polydextrose between 3 and 60 days of life. The use of probiotic or prebiotic from this same study significantly reduced the incidence of rhinovirus-associated infections which constituted 80.0% of all RTIs recorded in that study. Similarly, the incidence of viral RTI caused by the influenza virus was significantly reduced in a study involving 1,783 school children following the use of a probiotic, *L. brevis*.

A probiotic strain *B. longum* isolated from healthy Japanese infants showed antagonistic or anti-influenza type A virus, influenza virus (IFV) A/PR/8/34 (H1N1) activity through decreasing pro-inflammatory cytokines especially IL-6 and IFNγ, after 14 days oral administration. Furthermore, the use of *B. longum* significantly decreased symptoms associated with H1N1 infection [8]. Murosaki *et al*. [9] reported pro-inflammatory activity and a significant decrease in influenza virus type A (IVA-H1N1) titre from the lungs after the use of *L. plantarum*L-137 previously isolated from fermented foods. The combined use of *L. rhamnosus* CRL1505 and *L. rhamnosus* CRL1506 in respiratory syncytial virus (RSV) challenged mice significantly decreased the viral load with increased IFN-α stimulation and body weight [7]. In separate studies, *E. faecium* NCIMB 10415 and *L. plantarum* N4 individually showed antiviral activities against transmissible gastroenteritis coronavirus (TGEV), which causes severe disease with high mortality in young piglets [10]. Piglets fed with these probiotic strains had significantly lowered viral load with an enhanced immune response showing elevated expression of interleukin 6 and 8.

The prolonged persistence and isolation of SARS-CoV-2 from stools of infected patients even after recovery have, however, showed the likely involvement of the gut-lung crosstalk in COVID-19 pathogenesis. This further implies the active viability and replication of SARS-CoV-2 within the gut of infected persons. Intestinal microbial dysbiosis with an attendant decrease in the number of beneficial gut microorganisms including *Lactobacillus* and *Bifidobacterium spp*. have been reported among COVID-19 patients from a case series survey in China. Although several probiotic strains have been successively used for the treatment of gastrointestinal infections and the restoration of impaired intestinal microbiota, the use of probiotics in the control and/or prevention intestinal complications and dysbiosis as a result of SARS-CoV-2 infection becomes imperative. This will enhance prompt colonization, adaptation and proliferation hence, complementing the effects of other beneficial members of the microbiota and the restoration of normal flora.

With gradual detailed comprehension of SARS-CoV-2 pathogenesis and the involvement of gut microbiota, the use of probiotics therapy in the modulation of intestinal microbiota and the mitigation of COVID-19 and its associated comorbidities will be requisite in the holistic fight against the COVID-19 pandemic. Also, the strong correlations between the presence of certain probiotic strains belonging to *Corynebacterium* and *Dolosigranulum* genera especially *C. accolens, C. pseudodiphtheriticum* and *D. pigrum* with respiratory health make them promising probiotic candidates which can be used intranasally or as nasal spray probiotics to mitigate SARS-CoV-2 colonization of the respiratory tract and infection. Probiotics strains, *C. accolens, C. pseudodiphtheriticum* and *C. propinquum* have been reported to have broad-spectrum antimicrobial activity respiratory tract pathogens including against *S. pneumoniae, S. aureus* and *M. catarrhalis*, coagulase-negative staphylococci and different viruses [6].

Probiotic strains have been successfully used over the years to reduce colonization, incidence and severity of different intestinal and respiratory tract viral infections in both humans and animals. The use of probiotics as anti-COVID-19 will undoubtedly reduce the burden and severity of COVID-19 and its associated comorbidities thereby mitigating the pandemic. Due to the generally safe status of probiotics, large-scale probiotic trials can be initiated, funded and executed within a limited period without spending needless and prolonged-time waiting for generally safe and acceptable vaccines while COVID-19 rages on.
